# Central venous stenosis after subclavian versus internal jugular dialysis catheter insertion (CITES) in adults in need of a temporary central dialysis catheter: study protocol for a two-arm, parallel-group, non-inferiority randomised controlled trial

**DOI:** 10.1186/s13063-023-07350-9

**Published:** 2023-05-12

**Authors:** Ola Borgquist, Leila Naddi, Gracijela Božović, Matthias Hellberg, Martin Annborn, Fredrik Sjövall, Maria Adrian, Eva Hettinger, Pia Sjöberg, Thomas Kander

**Affiliations:** 1 Department of Clinical Sciences Lund, Lund University, Lund, Sweden; 2 Department of Cardiothoracic Surgery, Anaesthesia and Intensive Care, Skåne University Hospital, Lund, Sweden; 3 Department of Intensive and Perioperative Care, Skåne University Hospital, Lund, Sweden; 4 Department of Medical Imaging and Physiology, Skåne University Hospital, Lund, Sweden; 5 Department of Nephrology, Skåne University Hospital, Lund, Sweden; 6 Department of Anaesthesia and Intensive Care, Helsingborg Hospital, Helsingborg, Sweden; 7 Department of Intensive and Perioperative Care, Skåne University Hospital, Malmö, Sweden; 8 Department of Clinical Physiology, Skåne University Hospital, Lund, Sweden

**Keywords:** Central vein stenosis, Central venous stenosis, Subclavian vein, Internal jugular vein, Central dialysis catheter, Right supraclavicular fossa view, Randomised controlled trial

## Abstract

**Background:**

The right internal jugular vein is currently recommended for temporary central dialysis catheters (tCDC) based on results from previous studies showing a lower incidence of central vein stenosis compared to the subclavian vein. Data is however conflicting, and there are several advantages when the subclavian route is used for tCDCs. This prospective, controlled, randomised, non-inferiority study aims to compare the incidence of post-catheterisation central vein stenosis between the right subclavian and the right internal jugular routes.

**Methods:**

Adult patients needing a tCDC will be included from several hospitals and randomised to either subclavian or internal jugular vein catheterisation with a silicone tCDC. Inclusion continues until 50 patients in each group have undergone a follow-up CT venography. The primary outcome is the incidence of post-catheterisation central vein stenosis detected by a CT venography performed 1.5 to 3 months after removal of the tCDC. Secondary outcomes include between-group comparisons of (I) the patients’ experience of discomfort and pain, (II) any dysfunction of the tCDC during use, (III) catheterisation success rate and (IV) the number of mechanical complications. Furthermore, the ability to detect central vein stenosis by a focused ultrasound examination will be evaluated using the CT venography as golden standard.

**Discussion:**

The use of the subclavian route for tCDC placement has largely been abandoned due to older studies with various methodological issues. However, the subclavian route offers several advantages for the patient. This trial is designed to provide robust data on the incidence of central vein stenosis after silicone tCDC insertion in the era of ultrasound-guided catheterisations.

**Trial registration:**

Clinicaltrials.gov; NCT04871568. Prospectively registered on May 4, 2021.

**Supplementary Information:**

The online version contains supplementary material available at 10.1186/s13063-023-07350-9.

## Administrative information

**Table:** 

Title {1}	Central Venous Stenosis After Subclavian versus Internal Jugular Dialysis Catheter Insertion (CITES) in adults in need of a temporary central dialysis catheter: Study Protocol for a Two-Arm, Parallel-Group, Non-inferiority Randomised Controlled Trial
Trial registration {2a and 2b}	Clinicaltrials.gov (NCT04871568). Prospectively registered on May 4, 2021
Protocol version {3}	Version 1.1 April, 2023
Funding {4}	Southern Health Care Region Research funding, Sweden. Grant number: 2020–0230 (TK & OB) Lions Research Fund, Skåne, Sweden. No grant number (TK) The Swedish Society of Medicine. Grant number: SLS961142 (TK) The Kidney Foundation. No grant number (TK) Stig and Ragna Gorthons foundation. Grant number 2021–2791 (MAn)
Author details {5a}	Department of Clinical Sciences Lund, Lund University, Lund, Sweden (OB, LN, GB, MH, MAn, FS, MAd, EH, PS, TK) Department of Cardiothoracic Surgery, Anaesthesia and Intensive Care, Skåne University Hospital, Lund, Sweden (OB, MAd) Department of Intensive and Perioperative Care, Skåne University Hospital, Lund, Sweden (LN, TK) Department of Medical Imaging and Physiology, Skåne University Hospital, Lund, Sweden (GB, EH) Department of Nephrology, Skåne University Hospital, Lund, Sweden (MH) Department of Intensive and Perioperative Care, Skane University Hospital, Malmö, Sweden (FS) Department of Anaesthesia and Intensive Care, Helsingborg Hospital, Helsingborg, Sweden (MAn) Department of Clinical Physiology, Skåne University Hospital, Lund, Sweden (PS)
Name and contact information for the trial sponsor {5b}	Ola Borgquist, Department of Cardiothoracic Surgery, Anaesthesia and Intensive Care, Skåne University Hospital, 221 85 Lund, Sweden Thomas Kander, Department of Intensive and Perioperative Care, Skåne University Hospital, 221 85 Lund, Sweden
Role of sponsor {5c}	The current study is an investigator-initiated trial. Ola Borgquist and Thomas Kander are both sponsor investigators of the trial and are not only sponsoring the trial, but also conducting it as principal investigators. This means that they will be in charge of creating, coordinating, and carrying out the study.

## Introduction

### Background and rationale {6a}

Despite a lower incidence of infectious and thrombotic complications with subclavian vein catheters [1], the right internal jugular vein is currently recommended for temporary central dialysis catheter (tCDC) placement as it results in fewer catheter misplacements (1.4% vs 9.1% for the right subclavian vein [2]) and a lower incidence of central vein stenosis (CVS; 10% vs 42% for the subclavian veins [3]). CVS causes significant morbidity (e.g. increased number of catheter-related infections and earlier catheter removal) in haemodialysis patients [4] and subclavian vein stenosis will generally preclude the use of the entire ipsilateral arm for venous access.

There has been conflicting data regarding CVS incidence when comparing the subclavian and internal jugular veins [5–7]. An important shortcoming of most studies is that vascular imaging was performed for clinical indications which could have introduced a bias because patients without CVS are then more likely to be underrepresented. Furthermore, the definition of CVS varies between studies. A CT venography is deemed as a good modality since it is easily performed, readily available and time efficient [8]. It allows for visualisation of venous anatomy, patency and the presence of collaterals. Ultrasound, which is readily available in almost all intensive care units and recommended to be used during central venous catheterisation to increase success rates and decrease the numbers of mechanical complications [9], could also be used for systematic screening of patients to assess central vein patency before choosing the access site. Detection of venous stenosis may prevent attempts at central venous catheterisation that would be unsuccessful or could exacerbate the patient’s symptoms.

In addition to the previously described advantages of less infectious and thrombotic complications, the subclavian route may be more comfortable for the patient during insertion and also later on. Secondly, given that the subclavian route is further away from moving body parts compared to the internal jugular and the femoral routes, patient movement is less likely to affect the dialysis blood flow. Thirdly, the internal jugular and femoral routes are sometimes unavailable for catheterisation (e.g. due to local infection, thrombosis or other catheters in situ).

The catheter fabrication material (polyurethane vs silicone) may also have an impact on CVS incidence [6]. Polyurethane catheters (which were commonly used in previous studies) are stiffer and less compliant with the venous vessels and may thus have a greater impact on the vessel walls compared to softer silicone catheters.

To summarise, the subclavian route offers several advantages when placing dialysis catheters, but it is rarely used due to previous studies (with some methodological concerns) that have demonstrated a higher incidence of CVS compared to internal jugular catheterisation [10]. In an attempt to challenge this axiom, we designed the proposed two-arm, parallel-group, non-inferiority randomised controlled trial using silicone catheters inserted in the right subclavian or the right internal jugular vein.

### Objectives {7}

#### Hypothesis

After insertion and use of a silicone tCDC for ≥ 7 days, the incidence of post-catheterisation CVS is non-inferior when the right subclavian route is used as compared to the right internal jugular route.

#### Objectives

1. To determine if the incidence of post-catheterisation CVS detected by a CT venography performed 1.5–3 months after removal of the tCDC is non-inferior when the right subclavian route is used as compared to the right internal jugular route
2. To compare the performance of a focused ultrasound examination of the central veins to detect CVS with a CT venography (golden standard) with a threshold of
  a 50% venous diameter/area reduction (corresponding to moderate CVS in this study), as this is the commonly used definition of CVS [11]
  b 80% venous diameter/area reduction (corresponding to severe CVS in this study), as it has been suggested that the severity of central veno-occlusive disease must reach a diameter reduction around 80% before upstream changes in blood flow are detectable by current Doppler techniques [12]
3. To compare the patients’ experience of discomfort and pain between the groups
  a During the catheterisation, using a questionnaire (Additional file 1) handed out immediately after insertion
  b When carrying the catheter, using a questionnaire (Additional file 2) handed out as soon as possible after catheter removal
4. To compare the function of the tCDC during dialysis or plasmapheresis between the groups, using a questionnaire filled out by the dialysis nurse after each treatment session. The questionnaire (Additional file 3) includes both objective and subjective parameters
5. To compare the catheterisation success rate (defined as catheter tip placement in the right atrium or in the superior vena cava with the catheter aligned with the vessel) between the two groups using a post-procedural chest x-ray
6. To compare the number of mechanical complications (defined as arterial puncture/catheterisation and pneumothorax) between the groups

### Trial design {8}

This is a two-arm parallel-group, randomised controlled trial investigating whether the insertion of a dialysis catheter in the subclavian vein is non-inferior compared to the internal jugular vein with regard to the incidence of central venous stenosis.

## Methods: participants, interventions, and outcomes

### Study setting {9}

Patients will be recruited from at least five Swedish hospitals: Skåne University Hospital (Lund and Malmö), the county hospitals of Helsingborg and Jönköping, and Sahlgrenska University Hospital (Gothenburg).

### Eligibility criteria {10}

All patients that fulfil the inclusion criteria and lack all the exclusion criteria will be included in the study.

### Inclusioncriteria

1. Adult (≥ 18 years)
2. In need of a tCDC with an expected treatment time of at least 7 days
3. Informed consent

### Exclusioncriteria

1. Intravenous pacemaker or a PICC-line via right-sided central veins in situ
2. Known CVS
3. AV fistula in the right arm
4. History of central venous vascular interventions including stents, dilatations and more (but not previous central venous catheterisation)
5. Central venous catheter in the right internal jugular vein or in the right subclavian vein in situ
6. Either the right jugular vein or the right subclavian vein unavailable for catheterisation due to, e.g. local skin infection or thrombosis
7. Known allergy to iodinated contrast agents
8. BMI > 35 kg/m^2^
9. No study physician available for the catheterisation

### Who will take informed consent? {26a}

Patients will be identified and recruited by the inserting physician or by study personnel after referral for tCDC insertion. Both verbal and written study information will be given. Potential participants will be given the opportunity to ask questions about the study. Signed informed consent will then be obtained by a delegated research nurse or by the inserting physician.

### Additional consent provisions for collection and use of participant data and biological specimens {26b}

No additional consents are required. This trial does not involve collecting biological specimens for storage.

## Interventions

### Explanation for the choice of comparators {6b}

Previous studies that used stiffer polyurethane tCDCs and had different methodological issues have demonstrated higher incidence of CVS after subclavian catheterisation as compared to internal jugular catheterisation. As the subclavian route offers several advantages, the current study will investigate if the incidence of CVS after insertion of a soft silicone tCDC in the right-sided subclavian vein is non-inferior compared to the right internal jugular vein which, in clinical practice, is the vein most commonly used.

### Intervention description {11a}

All CDC-inserting physicians employed at the participating departments who have performed at least 100 ultrasound-guided central vein catheterisations (whereof at least 10 in the subclavian vein using the in-plane needling technique) will be asked to participate in the study as enrolling and inserting physicians. All participating physicians will certify that they fulfil these criteria in a signed curriculum vitae. To promote standardisation of the insertion procedure, all enrolling physicians must participate in an educational session on subclavian and internal jugular vein tCDC insertion.

### Coagulopathy

Coagulopathy does not affect the choice of catheterisation site and is thus of no importance with regard to study participation. Procoagulants can be administered at the discretion of the operator.

### Catheterisation protocol

Catheterisations will be performed in a standardised manner. A post-procedural chest x-ray is performed in every patient. Acceptable catheter tip locations are in the upper right atrium, at the cavo-atrial junction or in the superior vena cava with the catheter aligned with the vessel.

### Criteria for discontinuing or modifying allocated interventions {11b}

As the data will be analysed on a per protocol basis, patients that do not complete the follow-up for a specific outcome will not be included in the analyses of that outcome.

### Reasons to exit the trial

1. Inability to place the catheter in the randomised vein for any reason (the catheter may then be placed in any vein at the discretion of the operator)
2. Inability to achieve a correct catheter tip location
3. Patient desires to no longer take part in the trial

### Reasons for exclusion from primary outcome analyses as well as the secondary outcome concerning ultrasound examination

1. The patient is unable to perform a follow-up CT venography. Reasons for this include, but is not limited to
  a An estimated glomerular filtration rate < 15 ml/min without ongoing dialysis treatment at the time for the CT scan
  b The patient dies before follow-up
2. The tCDC is in situ for < 7 days
3. An additional central venous catheter is placed in the right-sided subclavian or internal jugular vein before the follow-up CT. Vein punctures without catheterisation are accepted (e.g. for myocardial biopsies after heart transplant surgery)

Reasons for exit from the trial and for exclusion in any of the analyses will be collected and reported. Data from the questionnaire about discomfort and pain during the catheterisation procedure will be used regardless and the questionnaire about discomfort and pain during the use of the tCDC will be used if the tCDC has been in situ for ≥ 7 days.

### Strategies to improve adherence to interventions {11c}

Given the nature of the intervention, no specific strategy is planned to improve adherence of the intervention.

### Relevant concomitant care permitted or prohibited during the trial {11d}

Restrictions are applied if additional central venous catheters are needed before the CT venography has been performed at 1.5–3 months after tCDC removal. Additional central venous catheters may not be placed in any of the study veins but rather in the left subclavian vein, the left internal jugular vein or in a femoral vein. To prevent protocol violations, the restriction will be clearly documented in the medical charts and patients will be informed about this.

### Provisions for post-trial care {30}

Provisions for post-trial care are not applicable.

### Outcomes {12}

The primary outcome is the incidence of post-catheterisation CVS, defined as venous diameter reduction > 50% anywhere from the estimated insertion site to the tip of the catheter, detected by a CT venography performed 1.5–3 months after removal of the tCDC.

The CT venography will be performed according to a protocol designed for this trial (Attachment 1) with the purpose to keep the amount of intravenous contrast and radiation as low as possible (with a limit of 100 kV the amount of intravenous contrast is reduced by 30% and less radiation is needed by repositioning the shoulders in an arm holder).

The secondary outcomes are:

1. To compare the performance of a focused ultrasound examination of the central veins to detect CVS with a CT venography (golden standard) with a threshold of
  a 50% venous diameter reduction, as this is the commonly used definition of CVS
  b 80% venous diameter reduction, as it has been suggested that the severity of central veno-occlusive disease must reach a diameter reduction around 80% before upstream changes in blood flow are detectable by current Doppler techniques
2. To compare the patients’ experience of discomfort and pain between the groups both during the catheterisation and when carrying the catheter by means of a questionnaire (Additional files 1 and 2)
3. To compare catheter blood flow during dialysis and reported catheter-related dialysis dysfunction between the groups. A questionnaire to address this will be filled out by the dialysis nurse after every treatment, including both objective and subjective measures (Additional file 3)
4. To compare the catheterisation success rate (defined as catheter tip placement in the superior vena cava or in the right atrium) between the two groups
5. To compare the number of mechanical complications (defined as arterial puncture/catheterisation and pneumothorax) between the groups

### Participant timeline {13}

See the enclosed flowchart (Additional file 4).

### Sample size {14}

Sample size calculation for the primary outcome was performed using an online calculator (https://www.sealedenvelope.com/power/binary-noninferior/) based on results of a previous study [3] that demonstrated a CVS incidence of 10% after tCDC insertion in the internal jugular vein. The rate of stenosis events in historical data has ranged from 10% for the jugular vein to 42% for the subclavian vein. Given the perceived and shown advantages with subclavian lines (less infections/thrombosis, more comfortable for the patient during insertion and use), we chose a 15% noninferiority margin as this is less than half the absolute difference, is judged to be clinically acceptable and results in a reasonable sample size. Calculations revealed that 50 patients per group are needed to be 80% sure that the upper limit of a one-sided 95% confidence interval (or equivalently a 90% two-sided confidence interval) will exclude a difference in favour of the standard group. In order to have 100 patients perform the CT venography for the per-protocol analysis, at least 200 patients will probably have to be included in the trial. Important differences found between the treatment groups (in terms of how many that did not complete the protocol) will be reported.

### Recruitment {15}

Patients will be recruited consecutively and will be identified by the doctor on call or by the dedicated nurse responsible for the tCDC insertion logistics at each site. As inclusion is dependent on an available study operator, each site will encourage CVC-inserting physicians to embrace the described technique and to participate in the educational session to fulfil the prerequisites to be able to enrol and insert tCDCs within the study.

## Assignment of interventions: allocation

### Sequence generation {16a}

Patients will be randomised and stratified for each centre. In addition, the randomisation will be performed in blocks with varying size (2, 4 and 6). The randomisation will be performed in REDCap (Research Electronic Data Capture, Nashville, TN, US), a browser-based, metadata-driven electronic data capture software [13] that will be used both for randomisation and as an eCRF.

### Concealment mechanism {16b}

None of the physicians performing the tCDC catheterisations will have access to the randomisation file.

### Implementation {16c}

The allocation sequence file will be generated by a researcher not affiliated to the project. Enrolment and assigning participants to interventions will be performed as previously described.

## Assignment of interventions: blinding

### Who will be blinded {17a}

The radiologist that assesses the primary outcome, i.e. interpreting CT venographies and the physician that performs the ultrasound/Doppler investigation in conjunction with the CT venography, will be blinded to study group allocation. In addition, the person who performs the statistical analyses will be blinded. The patient, the inserting physicians and other care providers are unable to be blinded.

### Procedure for unblinding if needed {17b}

Procedures for unblinding are not applicable for the current study.

## Data collection and management

### Plans for assessment and collection of outcomes {18a}

Electronic case report forms will be used for collection of all necessary data. Files showing the eCRFs used in the trial are available upon request.

### Plans to promote participant retention and complete follow-up {18b}

If needed, patients will receive an appointment at the department for nephrology after the tCDC removal. At this appointment, the patient will be assessed by the responsible nephrologist with regard to contraindications to perform a CT venography. Furthermore, the patient will be informed about the main benefit of performing a CT venography: mapping of the venous system, which is valuable when future need of central venous and/or dialysis access arises.

If a physical appointment is impossible or deemed unnecessary, a nephrologist or a dedicated study physician will review patient records and labs to ensure there are no contraindications to perform a CT venography.

Regarding outcomes that may be collected for patients that do not complete the follow-up, please see the “Criteria for discontinuing or modifying allocated interventions {11b} section.”

### Data management {19}

Randomisation and data collection will be performed using an eCRF within REDCap, a secure web application. Randomisation, data collection and entry in the eCRF are performed both by dedicated research nurses and by the investigators. Data quality is promoted in the eCRF, e.g. by range checks. Paper forms are used for patient questionnaires and dialysis protocols and will be scanned into the eCRF by dedicated research nurses and later transferred to the main database.

### Confidentiality {27}

All data will be stored and handled according to the Swedish data protection regulation. Study participants will be identified by a study number in REDCap to ensure confidentiality. The code linking a study number to a specific patient will be stored in a computer in a secure location within the hospital premises. REDCap access will be restricted to site investigators and research nurses. Anonymous trial data may be shared with other researchers in the future to enable international prospective meta-analyses.

### Plans for collection, laboratory evaluation and storage of biological specimens for genetic or molecular analysis in this trial/future use {33}

No biological specimens will be collected in the current study.

## Statistical methods

### Statistical methods for primary and secondary outcomes {20a}

The study aims to explore if the incidence of post-catheterisation CVS when using the right-sided subclavian route is non-inferior to the internal jugular route. All patients randomised in this trial will be analysed on a per-protocol basis. The primary outcome will thus be analysed only in patients subjected to the follow-up CT venography. Patients will be enrolled using previous exit rates to determine when the calculated sample size for both groups can be reached.

### Baseline variables

Continuous variables following a symmetric distribution will be presented with mean (SD), while skewed variables will be presented with median (IQR). Ordinal variables will be presented with median (IQR) and categorical variables will be presented as *n* (%).

The primary outcome will be analysed as a binary variable (stenosis *vs* no stenosis) at follow-up and described using a 95% one-sided confidence interval for the absolute difference between the proportions, calculated as event rate intervention group − event rate control group. If the upper limit of the confidence interval is below the non-inferiority margin, the conclusion will be that the event rate in the intervention group is non-inferior to the event rate in the control group.

### Interim analyses {21b}

No interim analyses are planned for the current study.

### Methods for additional analyses (e.g. subgroup analyses) {20b}

As the sample size is not very large, there is a risk for imbalance in baseline characteristics between groups. This possible imbalance will be tested in a sensitivity test using univariate analyses. If needed, a comparison of the primary outcome between the groups will then be performed with a test where baseline characteristics can be stratified (e.g. the Cochran-Mantel–Haenszel test).

The Wilcoxon rank-sum test will be used to examine if the time until the CT venography differs between the groups.

Evaluation of the US/Doppler method will be made only in patients having undergone both an US/Doppler examination and CT venography. Sensitivity and specificity for the US-based method as compared to the golden standard, CT venography, will be determined and presented along with 95% confidence intervals. This will be performed for both intervention groups pooled together.

For all other secondary outcomes (the patients’ experience of the catheterisation, catheter blood flow during dialysis, catheter-related dialysis dysfunction, mechanical complications), data are expressed as mean (SD) or median and range. Qualitative variables are expressed as frequency with percentage. The *χ*^2^ test (or Fisher’s exact test as appropriate) will be used for comparison of categorical variables whereas the Student’s *t*-test and the Mann–Whitney *U* test will be used to compare continuous variables with normal and skewed distributions, respectively. A type 1 error rate of 5% will be used as threshold for statistical significance.

### Methods in analysis to handle protocol non-adherence and any statistical methods to handle missing data {20c}

Missing data will be reported in the publication and if it is not valid to ignore missing data we will consider computing best–worst and worst-best case scenarios.

### Plans to give access to the full protocol, participant level-data and statistical code {31c}

All data and statistical codes will be available upon reasonable request.

## Oversight and monitoring

### Composition of the coordinating centre and trial steering committee {5d}

The trial steering committee, including representatives from all participating departments, is anticipated to meet quarterly during the trial. The committee does not contain independent members who are not participating. Dedicated research nurses will assist the trial on a day-to-day basis.

### Composition of the data monitoring committee, its role and reporting structure {21a}

Data monitoring will be performed by an independent data monitoring committee (DMC) that consists of members of the Clinical Research Unit, Skåne University Hospital, in Lund.

### Frequency and plans for auditing trial conduct {23}

Each site will have monitoring done at three occasions: before study start, early during the trial (after approximately 10 patients have been included at the site) and when the last patient has performed the CT venography. Dedicated research nurses, without co-authorship in the current study, will monitor the data inserted in the eCRF on a monthly basis.

The trial steering group will meet at least every second month to review trial conduct. The trial steering group and the DMC meet to review conduct throughout the trial period.

### Adverse event reporting and harms {22}

Any event that has a reasonable, causal relationship to the study intervention will be deemed an adverse event (AE). All patients are evaluated with regard to potential AEs or serious adverse events (SAE) by one of the investigators. Potential AEs and SAEs are recorded in the eCRF and should be hastily reported to the chief investigators for decisions on any change in the protocol. Furthermore, the DMC will be notified whenever there is a serious adverse event.

### Plans for communicating important protocol amendments to relevant parties (e.g. trial participants, ethical committees) {25}

Important changes in this protocol will be reported to the National Ethical Review Board, Sweden, and an amendment approval will be sought. Further, important changes will be registered in ClinicalTrials.gov and communicated to the site investigators, research nurses and monitors by established networks.

### Dissemination plans {31a}

The main manuscript describing the results of the current trial will be submitted to a peer-reviewed journal regardless of the results of the trial. Authorship will be determined in accordance with the International Committee of Medical Journal Editors and detailed criteria needed for co-authorship will be decided in advance.

## Discussion

The CITES trial is designed to provide robust data on whether the incidence of CVS after insertion of a silicone tCDC in the right subclavian vein is non-inferior compared to when using the internal jugular vein. This is clinically important as the subclavian route offers several advantages for the patient (e.g. less infectious and thrombotic complications) and because other routes are sometimes unavailable for catheterisation.

The current study is not an evaluation of a clinical routine but rather aims to explore the proof of concept that the subclavian route is non-inferior to the internal jugular route with regard to the incidence of post-catheterisation CVS. This design is based on the knowledge that older studies [3, 14] were performed with stiff polyurethane catheters and had methodological issues such as lack of randomisation, varying definitions of CVS and selection bias (e.g. only patients with a clinical indication for CVS investigation were included).

To allow for thrombosis and any temporary narrowing of central veins to resolve, a time interval for the CT venography of 1.5–3 months after removal of the tCDC was chosen.

Data from the era of landmark-based catheterisations have indicated less misplacements when using the left-sided subclavian vein [15]. However, right-sided catheterisation was chosen for this study as it allows for having the supraclavicular fossa view within the sterile field, which makes real-time ultrasound-guided navigation of the guidewire tip possible [16]. Furthermore, the right internal jugular vein is the vein of choice for dialysis catheters [17], right-sided catheters are generally 5 cm shorter than the left-sided ones which will allow for less resistance to blood flow [18] and less catheter area is exposed to the veins.

The administration of intravenous contrast within the study is done in accordance with the European Society of Urogenital Radiology (ESUR) guidelines [19]. Thus, patients with severe renal failure (GFR < 15 mL/min/1.73 m^2^) without ongoing dialysis at follow-up will be excluded from the study as intravenous contrast can affect the renal function in patients with renal impairment [14]. The amount of contrast and the radiation dose administered to the patient have been minimised in the current protocol.

Real-time ultrasound guidance is used in > 93% of central venous catheterisations at the hospitals within Region Skåne (unpublished data). Thus, it would seem prudent to also make use of the ultrasound equipment to verify that the central veins are patent prior to the procedure to save time and avoid added patient discomfort. In order to gain acceptance and allow for easy implementation, the protocol is kept simple (Additional file 5).

## Trial status

Recruitment started on November 15, 2021, and is anticipated to end by December 2025.

## Supplementary Information

**Additional file 1.** Questionnaire handed out immediately after insertion of the catheter.

**Additional file 2.** Questionnaire handed out as soon as possible after catheter removal.

**Additional file 3.** Questionnaire which includes both objective and subjective parameters.

**Additional file 4.** Enclosed flowchart.

**Additional file 5.** Protocol

## Data Availability

This document constitutes the full protocol. Following the completion of the trial datasets used in this study, it will be available from the corresponding author on reasonable request.

## Abbreviations

AV fistula: Arteriovenous fistula
BMI: Body mass index
CT: Computed tomography
CVS: Central vein stenosis
PICC-line: Peripherally inserted central catheter line
tCDC: Temporary central dialysis catheter
