# Supplementary material for: Central venous stenosis after subclavian versus internal jugular dialysis catheter insertion (CITES) in adults in need of a temporary central dialysis catheter: study protocol for a two-arm, parallel-group, non-inferiority randomised controlled trial
Source: Trials. 2023 May 12;24:327. doi: 10.1186/s13063-023-07350-9 (PMC10176902; doi:10.1186/s13063-023-07350-9)
Supplement: Supplementary file 1 — Additional file 1. Questionnaire handed out immediately after insertion of the catheter. [file 13063_2023_7350_MOESM1_ESM.pdf]

Patient survey central dialysis catheter insertion

Study number: ______

Name: ______

Date: ___________

What **degree of discomfort** did you experience when the dialysis catheter was inserted?	**0**	**1**	**2**	**3**	**4**	**5**	**6**	**7**	**8**	**9**	**10**
	No discomfort									Worst possible discomfort	
What **degree of pain** did you experience when the dialysis catheter was inserted?	**0**	**1**	**2**	**3**	**4**	**5**	**6**	**7**	**8**	**9**	**10**
	No pain									Worst possible pain	

Did you experience anything else that was stressful when the catheter was inserted?

Did you experience anything else that was helpful or facilitated when the catheter was inserted?
