# Supplementary material for: Central venous stenosis after subclavian versus internal jugular dialysis catheter insertion (CITES) in adults in need of a temporary central dialysis catheter: study protocol for a two-arm, parallel-group, non-inferiority randomised controlled trial
Source: Trials. 2023 May 12;24:327. doi: 10.1186/s13063-023-07350-9 (PMC10176902; doi:10.1186/s13063-023-07350-9)
Supplement: Supplementary file 2 — Additional file 2. Questionnaire handed out as soon as possible after catheter removal. [file 13063_2023_7350_MOESM2_ESM.pdf]

Patient survey wearing the central dialysis catheter

Study number: ______

Name: ____

Date: ___________________

Statement	0	1	2	3	4	5	6	7	8	9	10
When I got out of bed I experienced discomfort/pain from the catheter	0 Never	1	2 Rarely	3	4	5 Sometimes	6	7 Often	8	9	10 Always
When I moved in my bed I experienced discomfort/pain from the catheter	0 Never	1	2 Rarely	3	4	5 Sometimes	6	7 Often	8	9	10 Always
When I got dressed I experienced discomfort/pain from the catheter	0 Never	1	2 Rarely	3	4	5 Sometimes	6	7 Often	8	9	10 Always
I did not experience discomfort/pain, but the localisation of the catheter disturbed me	0 Never	1	2 Rarely	3	4	5 Sometimes	6	7 Often	8	9	10 Always
I was careful when moving my arm	0 Never	1	2 Rarely	3	4	5 Sometimes	6	7 Often	8	9	10 Always
I was careful when moving my head	0 Never	1	2 Rarely	3	4	5 Sometimes	6	7 Often	8	9	10 Always
I was happy with the localisation of my catheter	0 Never	1	2 Rarely	3	4	5 Sometimes	6	7 Often	8	9	10 Always
I experienced discomfort/pain when the catheter was removed	0 No discomfort/pain	1	2	3	4	5	6	7	8	9	10 Worst possible pain/discomfort

Did you experience anything else that was problematic and/or caused distress regarding wearing the catheter?
