# Supplementary material for: Central venous stenosis after subclavian versus internal jugular dialysis catheter insertion (CITES) in adults in need of a temporary central dialysis catheter: study protocol for a two-arm, parallel-group, non-inferiority randomised controlled trial
Source: Trials. 2023 May 12;24:327. doi: 10.1186/s13063-023-07350-9 (PMC10176902; doi:10.1186/s13063-023-07350-9)
Supplement: Supplementary file 3 — Additional file 3. Questionnaire which includes both objective and subjective parameters. [file 13063_2023_7350_MOESM3_ESM.pdf]

Protocol for catheter function during dialysis/plasmapheresis for patients in the CITES trial

Patient name: ______________________________

Patient personal id no: ______________________________

Patient study no: ______________________________ *Noted by study nurse or study physician*

Dialysis ☐ For dialysis *all* parameters are noted

Plasmapheresis ☐ For plasmapheresis only parameters in **bold style** are noted

Date (for each treatment)				
Objective measurements				
Flow test ok?*	YES / NO	YES / NO	YES / NO	YES / NO
If flow test *not* ok, which was the highest possible blood flow? (ml/min)				
Max arterial pressure during flow test (mm Hg)				
Max venous pressure during flow test (mm Hg)				
**Was it possible to carry out the prescribed treatment?**	**YES / NO**	**YES / NO**	**YES / NO**	**YES / NO**
**Was the CDC location dependent?**	**YES / NO**	**YES / NO**	**YES / NO**	**YES / NO**
Was the treatment carried out with reversed flow?	YES / NO	YES / NO	YES / NO	YES / NO
Subjective measurements				
Easy to aspirate from the CDC?	YES / NO	YES / NO	YES / NO	YES / NO
Easy to flush the CDC?	YES / NO	YES / NO	YES / NO	YES / NO
How well did the CDC work during the treatment? Please note a number between 0 and 10, with 0 meaning *not well at all* and 10 meaning *very well*.				

Circle YES *or* NO for each question

*Flow test = blood flow 250 ml/min during 15 mins. Flow test is performed after about 1h into the dialysis treatment.

When patient no longer is planned for more treatments, all *Protocol for catheter function* are collected together with copies of all *Dialysis protocol* + copy of *Approval of central dialysis catheter* and placed in designated binder at the dialysis ward, where study physician Leila Naddi will collect them

If the CDC is removed at the dialysis ward, please send notification in Melior to research nurse Susann Schrey, Intensive and perioperative care.

Any questions or need more protocols, please contact study physician Leila Naddi, phone no 73699 or leila.naddi@skane.se THANK YOU FOR YOUR HELP!
