# Supplementary material for: Central venous stenosis after subclavian versus internal jugular dialysis catheter insertion (CITES) in adults in need of a temporary central dialysis catheter: study protocol for a two-arm, parallel-group, non-inferiority randomised controlled trial
Source: Trials. 2023 May 12;24:327. doi: 10.1186/s13063-023-07350-9 (PMC10176902; doi:10.1186/s13063-023-07350-9)
Supplement: Supplementary file 4 — Additional file 4. Enclosed flowchart. [file 13063_2023_7350_MOESM4_ESM.pdf]

Flowchart for the CITES trial

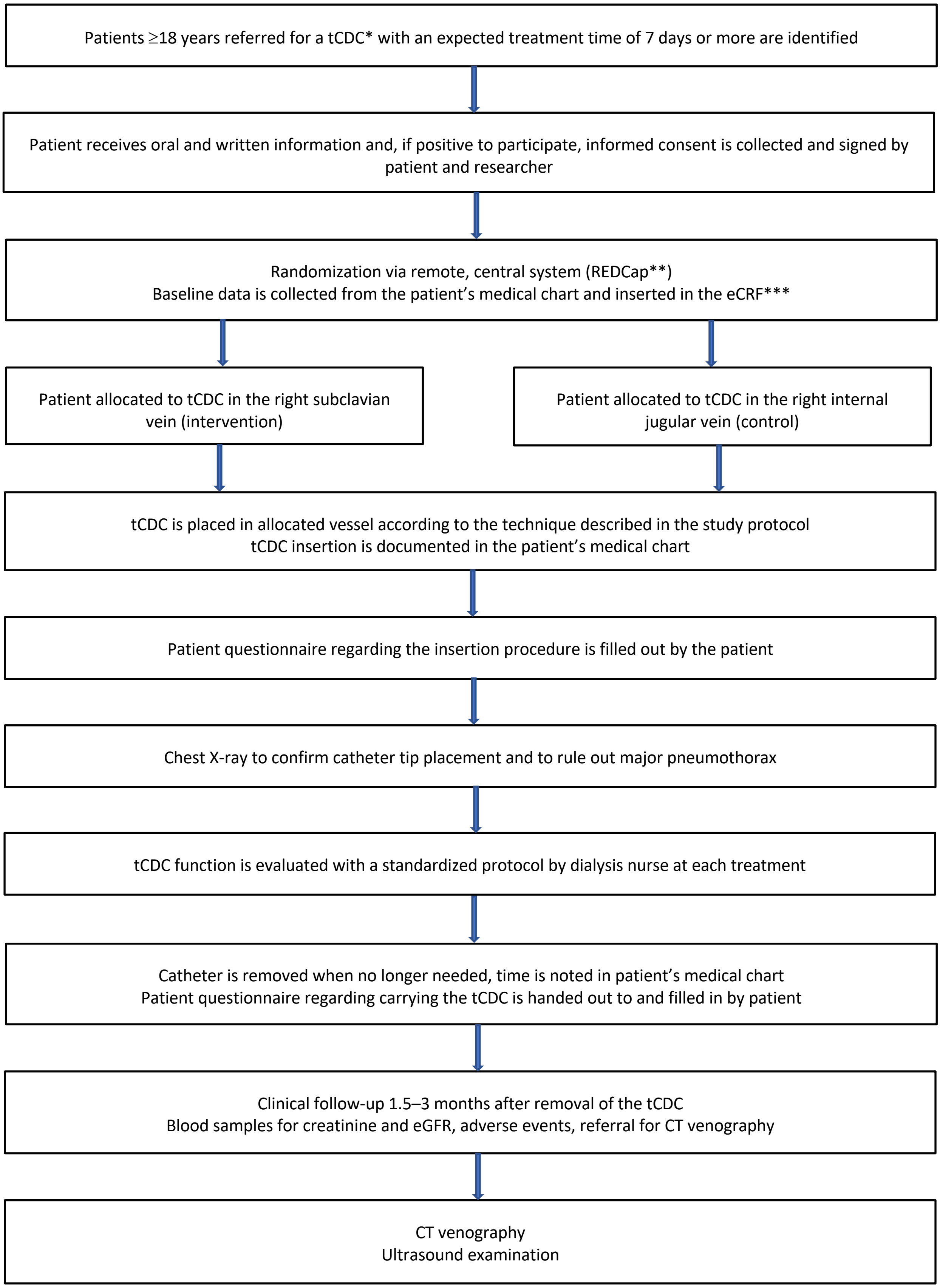

*temporary dialysis catheter **Research Electronic Data Capture ***electronic case report form
