# Supplementary material for: Central venous stenosis after subclavian versus internal jugular dialysis catheter insertion (CITES) in adults in need of a temporary central dialysis catheter: study protocol for a two-arm, parallel-group, non-inferiority randomised controlled trial
Source: Trials. 2023 May 12;24:327. doi: 10.1186/s13063-023-07350-9 (PMC10176902; doi:10.1186/s13063-023-07350-9)

Ultrasound protocol for assessment of CVS

The aims for the US scan are to verify vein patency and to detect CVS.

General considerations

- ☐ The study is performed in the supine position.
- ☐ The head is placed in a neutral position.
- ☐ Quiet respiration is preferred – provocative maneuvers such as Valsalva are not employed.
- ☐ The least possible pressure is applied on the probe to avoid compressing the vessels.

Assessment of superior vena cava, the right internal jugular, the right subclavian and the right brachiocephalic veins

- ☐ The vein is assessed with regards to *visible thrombus*.
- ☐ The *filling pressure* of the vein is determined to be “distended”, “regular” (normal size but collapses with pressure) or “hypovolemic” (small, easily collapsible, marked variation with respiration).
- ☐ A *Doppler examination* is performed in a long axis view of the vein.
    - ☐ The Doppler sample volume is set at the smallest size possible and placed at the center of the vessel.
    - ☐ The gain is appropriately set.
    - ☐ An angle of 60° or less as measured between insonation beam and blood flow direction or vessel wall is used.
    - ☐ *Respiratory phasicity* is seen as a “surge” in the venous flow due to an increase in flow during inspiration. The presence of stenosis and/or an occlusion in the central veins (subclavian vein, brachiocephalic trunk and superior cava vein) will cause the absence of this wave.
    - ☐ C*ardiac pulsatility* is due to the closure of the heart valves which exerts a pressure wave that causes small oscillations in the Doppler curve coinciding with the cardiac cycle. These oscillations are not seen in cases of stenosis or occlusion of the central veins.

Definition of CVS based on ultrasound and Doppler investigations

A patient fulfilling any of these criteria is considered to have a CVS:

- ☐ A visible diameter reduction >50%.
- ☐ A dampened or continuous waveform without a pulsatile flow pattern (see below).

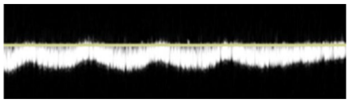
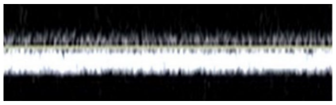

Supplement: Supplementary file 5 — Additional file 5. Protocol [file 13063_2023_7350_MOESM5_ESM.pdf]
